# Absence of susceptibility vessel sign and hyperdense vessel sign in patients with cancer-related stroke

**DOI:** 10.3389/fneur.2023.1148152

**Published:** 2023-03-20

**Authors:** Morin Beyeler, Lorenz Grunder, Jayan Göcmen, Fabienne Steinauer, Nebiyat F. Belachew, Moritz Kielkopf, Leander Clénin, Madlaine Mueller, Norbert Silimon, Christoph Kurmann, Thomas Meinel, Philipp Bücke, David Seiffge, Tomas Dobrocky, Eike I. Piechowiak, Sara Pilgram-Pastor, Heinrich P. Mattle, Babak B. Navi, Marcel Arnold, Urs Fischer, Thomas Pabst, Jan Gralla, Martin D. Berger, Simon Jung, Johannes Kaesmacher

**Affiliations:** ^1^Department of Neurology, Inselspital, Bern University Hospital, and University of Bern, Bern, Switzerland; ^2^Graduate School for Health Sciences, University of Bern, Bern, Switzerland; ^3^Institute for Diagnostic and Interventional Neuroradiology, Inselspital, Bern University Hospital, and University of Bern, Bern, Switzerland; ^4^Department of Neuroradiology, University Hospital, Freiburg, Germany; ^5^Clinical and Translational Neuroscience Unit, Feil Family Brain and Mind Research Institute and Department of Neurology, Weill Cornell Medicine, New York, NY, United States; ^6^Neurology Department, University Hospital of Basel, University of Basel, Basel, Switzerland; ^7^Department of Medical Oncology, Inselspital, Bern University Hospital, and University of Bern, Bern, Switzerland

**Keywords:** cancer-related stroke, thrombus imaging characteristics, susceptibility vessel sign, hyperdense vessel sign, malignancy-related stroke

## Abstract

**Background and aim:**

Identification of paraneoplastic hypercoagulability in stroke patients helps to guide investigations and prevent stroke recurrence. A previous study demonstrated an association between the absence of the susceptibility vessel sign (SVS) on brain MRI and active cancer in patients treated with mechanical thrombectomy. The present study aimed to confirm this finding and assess an association between the absence of the hyperdense vessel sign (HVS) on head CT and active cancer in all stroke patients.

**Methods:**

SVS and HVS status on baseline imaging were retrospectively assessed in all consecutive stroke patients treated at a comprehensive stroke center between 2015 and 2020. Active cancer, known at the time of stroke or diagnosed within 1 year after stroke (occult cancer), was identified. Adjusted odds ratios (aOR) and their 95% confidence interval (CI) for the association between the thrombus imaging characteristics and cancer were calculated using multivariable logistic regression.

**Results:**

Of the 2,256 patients with thrombus imaging characteristics available at baseline, 161 had an active cancer (7.1%), of which 36 were occult at the time of index stroke (1.6% of the total). The absence of SVS was associated with active cancer (aOR 3.14, 95% CI 1.45–6.80). No significance was reached for the subgroup of occult cancer (aOR 3.20, 95% CI 0.73–13.94). No association was found between the absence of HVS and active cancer (aOR 1.07, 95% CI 0.54–2.11).

**Conclusion:**

The absence of SVS but not HVS could help to identify paraneoplastic hypercoagulability in stroke patients with active cancer and guide patient care.

## Introduction

Cancer-related strokes are generally more severe than other stroke types and associated with an increased risk of stroke recurrence and poor outcomes (1, 2). Therefore, it is essential to identify them and initiate timely and adequate secondary prevention (3). Furthermore, earlier detection of occult cancer (defined as unknown cancer with ischemic stroke as first manifestation) in stroke patients would allow more rapid treatment of cancer, which could improve patient outcomes (4–7). Cancer-related strokes are often caused by paraneoplastic hypercoagulability and associated with abnormal coagulation and blood parameters [such as elevated D-dimer and C-reactive protein (CRP), lower hemoglobin (Hb)] as well as multiterritorial infarcts (8–10). In addition, fibrin and platelet-rich thrombi retrieved during mechanical thrombectomy are associated with cancer in stroke patients (11, 12). The composition of intracranial thrombi can be assessed non-invasively and *in situ* with thrombus imaging characterization as a surrogate marker (13). Our previous study demonstrated an association between the absence of the susceptibility vessel sign (SVS) in susceptibility-weighted imaging (SWI) on brain magnetic resonance imaging (MRI) and active cancer in patients treated with mechanical thrombectomy (14). The reason for the absence of SVS with active cancer may be due to a predominance of fibrin and platelets and a relative paucity of erythrocytes in these patients thrombus composition (11, 12, 15). The present study aimed to validate the association between the absence of SVS and active cancer in all stroke patients. Furthermore, we aimed to evaluate an association between active cancer and the absence of the hyperdense vessel sign (HVS) in native computed tomography (CT) as the absence of HVS has also been shown to be associated with fibrin and platelet-rich thrombi (16, 17).

## Methods

### Study population

This retrospective cohort study comprised consecutive patients diagnosed with acute ischemic stroke from January 1, 2015 to December 31, 2020 who were entered into our prospective institutional stroke registry. Study inclusion criteria were: (1) an acute ischemic stroke with the presence of at least one symptomatic intracranial arterial occlusion, (2) available acute imaging for review from an internal or external (before referral) baseline MRI brain scan with SWI sequences or CT head scan with standard sequences, and (3) sufficient quality of an acute baseline brain imaging sequences to assess for SVS and/or HVS (study flowchart – Supplementary Figure S1). Patients who underwent intravenous thrombolysis (IVT) before available baseline imaging were excluded. Additionally, when IVT was administered after baseline imaging but before blood examination, these patients were excluded from a secondary analysis involving blood biomarkers. The ethics committee approved the study’s conduct in accordance with Swiss law (reference ID: 2021-01031, Kantonale Ethikkomission Bern). According to the ethics committee’s decision, no informed consent was required for the inclusion of patients in the study.

### Definition of active cancer and occult cancer

Active cancer was identified according to the definition from the Haemostasis and Malignancy Scientific and Standardization Committee of the International Society on Thrombosis and Haemostasis (18, 19). Newly diagnosed cancer within one year after the index stroke was defined as “occult cancer” at the time of stroke and considered an active cancer for the purposes of this study (4, 20, 21). As defined previously, patients with focal non-melanoma skin cancer and those treated with prophylactic hormone therapy for prior breast cancer were classified as not having active cancer (14, 22, 23).

### Imaging analysis

**Figure 1:**
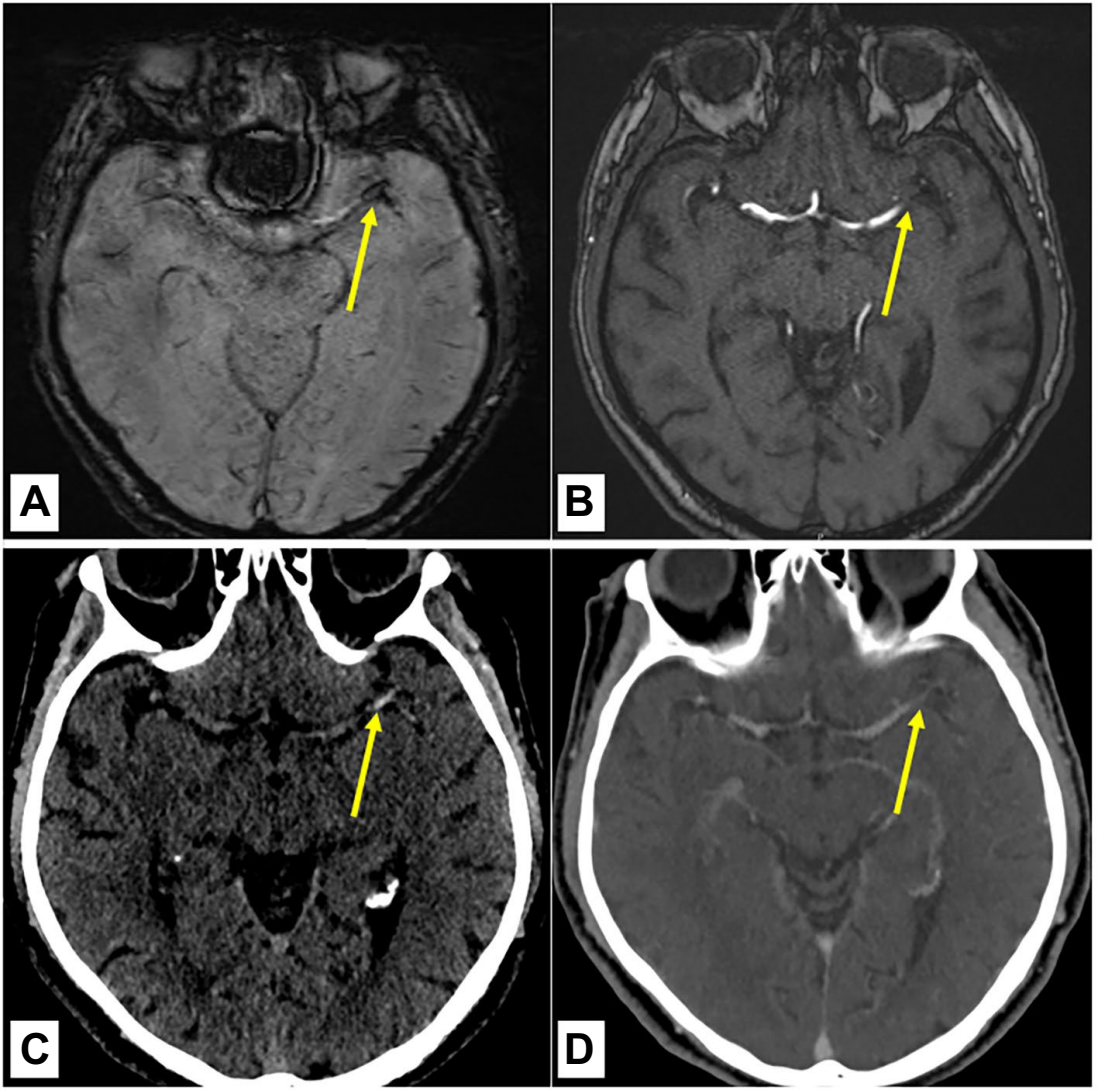
Assessment of the susceptibility vessel sign and hyperdense vessel sign status on baseline brain imaging. **(A,B)** 70-year-old male patient with an acute ischemic stroke due to the occlusion of the left MCA (M1 segment) diagnosed on baseline brain MRI with present SVS (arrowhead) as local hypointensity on the SWI **(A)** with confirmation of occlusion on the arterial TOF **(B)**. **(C,D)** Externally performed CT imaging of the same patient before referring for thrombectomy. Presence of HVS (arrowhead) as local hyperdensity in native CT **(C)** with confirmation of occlusion in the CT angiography **(D)**. Yellow arrows point to the proximal part of the vessel occlusion. HVS indicates hyperdense vessel sign; MCA, middle cerebral artery; SVS, susceptibility vessel sign; SWI, susceptibility weighted imaging and TOF, time of flight angiography.

Acute imaging was performed on a 1.5 T or 3 T MR imaging scanner (Magnetom Avanto, Magnetom Aera, Magnetom Verio and Magnetom Vida; Siemens, Erlangen, Germany). 1.5 T SWI was performed with the following parameters: Repetition time (TR), 49 ms; echo time (TE), 40 ms; flip angle, 15.0°; section thickness, 1.6, 1.8, or 2.0 mm; and intersection gap, 0 mm. 3 T SWI was performed with the following parameters: TR, 27 ms; TE, 20 ms; flip angle, 15.0°; section thickness, 2.0 mm; and intersection gap, 0 mm. Standard native CT was performed on a 128-row CT scanner (Siemens SOMATOM Edge, Siemens Erlangen, Germany) by use of CarekV (Quality reference of 120 kV), modulated milliampere-seconds (mAs) with CareDose4D (Quality reference of 290 mAs), with 1.0 mm section thickness. CT angiography consisted of 0.6 mm slice thickness bolus-triggered acquisition, 1 mm slice thickness late venous acquisition with a 75-s delay after bolus administration. The side and site of intracranial arterial occlusion as well as the SVS and HVS status were assessed by a stroke neurologist and a neuroradiologist (M.B. and L.G.). Both raters were blinded to baseline characteristics and clinical outcomes. The SVS status was assessed according to the method described previously by Belachew et al. (24) SVS was considered to be present if signal loss corresponded to the symptomatic intracranial occlusion and no alternative reason existed (Figures 1A,B). HVS status was determined intrinsically on native CT at the location directly corresponding to the occlusion site on CT angiography. HVS was considered present in the case of local hyperdensity by co-locating the occluded artery with its contralateral homologue and the surrounding brain tissue (Figures 1C,D) (25). Interrater reliability between investigators, as determined by Cohen’s Kappa coefficient, was sustainable (0.770 for SVS and 0.610 for HVS). As HVS is more frequent in proximal than distal occlusions and the imaging characterization of thrombi is more accurate in proximal occlusion, we distinguished between proximal and distal occlusions for subgroup analysis (26). Visible vessel occlusions at baseline were assessed on CT angiography, time-of-flight MR angiography or contrast-enhanced MR angiography. Proximal occlusions were defined as intracranial occlusion of the internal carotid artery, M1 and M2 segments of the middle cerebral artery, A1 segment of the anterior cerebral artery, P1 segment of the posterior cerebral artery, vertebral artery and any segment of the basilar artery. Other occlusions were categorized as distal.

### Data collection

Demographics and baseline stroke characteristics were extracted from the local stroke registry. This included gender, age, prestroke independence (modified Rankin Scale ≤2), admission blood pressure, prior treatment with anticoagulants, antiplatelet drugs and lipid-lowering drugs, cardiovascular risk factors, National Institutes of Health Stroke Scale (NIHSS) score on admission, time from last-known-well to admission and imaging (LKW-imaging), IVT, acute baseline imaging modality from the referral center or at admission at the enrolling center and laboratory values at admission: glucose in mmol/L, low-density lipoprotein (LDL) cholesterol in mmol/L, total cholesterol in mmol/L, albumin in g/L, lactate dehydrogenase (LDH) in U/L, D-dimer in μg/L, Hb in g/L, CRP in mg/L, leukocytes in G/L, thrombocytes in G/L, fibrinogen in g/L and international normalized ratio (INR). Two neurology fellows (J.G. and F.S.), blinded to the thrombus imaging characterization, retrospectively identified patients with active cancer (known or occult cancer) at the time of stroke by reviewing discharge, follow-up reports or histological findings available in medical records. In the case of active cancer, we extracted data on type, histology and time of diagnosis. Stroke etiology at discharge was determined using the Trial of ORG 10172 in Acute Stroke Treatment (TOAST) classification (27). Patent foramen ovale related strokes were classified as cardioembolic strokes according to recommended updated criteria (28). A subgroup of patients with undetermined etiology and non-lacunar ischemic stroke pattern on imaging were classified as embolic stroke of undetermined source (ESUS), according to published criteria (29, 30). The presence of multiterritory infarcts (i.e., involving 2 or more brain vascular territories) was identified through evaluation of baseline neuroradiological reports and imaging (10).

### Statistical analysis

Baseline differences between patients with and without active cancer were assessed for categorical variables with the Fisher’s exact test and reported as absolute numbers and proportions. Continuous variables were assessed with the Mann–Whitney *U*-test and reported as median with interquartile range (IQR). Same analyses were performed regarding the absence or presence of SVS and HVS, respectively. Univariable and multivariable logistic regression were used to assess the association between thrombus imaging characterization (SVS and HVS, separately) and active cancer. Odds ratios (ORs) and adjusted odds ratios (aORs) were reported with their corresponding 95% confidence intervals (95% CI). All models were adjusted for baseline characteristics (gender, age at admission, prior treatment with anticoagulants and antiplatelet drugs), proximal occlusion, LKW-imaging, ESUS and cancer-related biomarkers such as multiterritory infarcts, CRP, D-dimer, fibrinogen, Hb and leukocytes. Skewed distributions of continuous variables were logarithmically transformed. The predictive values of SVS and HVS were assessed using sensitivity, specificity, positive predictive value (PPV), negative predictive value (NPV), positive likelihood ratio (LR+), and negative likelihood ratio (LR−). When both imaging modalities (native CT + MRI with SWI) were available and had been acquired before initiating IVT, intermodal change in the thrombus imaging characterization was assessed. Sensitivity analyses were performed for (1) proximal versus distal occlusions, (2) time delays between last-known-well and imaging (LKW-imaging as a continuous value), (3) presence of ESUS at discharge and (4) occult cancer. Areas under the receiver operating characteristics curve (auROC) were calculated for predictive models including and excluding the SVS and the HVS status. After cross-validation using bootstrapping, the auROCs were compared using the Delong test. No imputation was applied to account for missing data. Statistical analyses were performed with Stata 16 (StataCorp LLC).

## Results

### Study population

Between 2015 and 2020, 5,012 patients with acute ischemic stroke were included in our local stroke registry. At baseline, 2509 of these patients had a symptomatic intracranial occlusion (study flowchart – Supplementary Figure S1). Thrombus imaging status in acute care setting was assessable in 2256 patients (1,175 with SVS status, 980 with HVS status and 101 with SVS and HVS status available) who were consequently included in the study. The distribution of occlusion sites is summarized in Supplementary Figure S2. Active cancer at the time of stroke was present in 161 included patients (7.1%) and occult cancer in 36 patients (1.6%). The characteristics of active cancers and occult cancers are summarized in Supplementary Figure S3. IVT treatment before the time of blood examination led to the exclusion of 298 patients (13.2%) from analyses involving blood biomarkers.

### Baseline characteristics

The characteristics of patients with and without active cancer are compared in Supplementary Table S1. Patients with active cancer showed multiterritorial infarcts more frequently, and usually presented with higher D-dimer, CRP, LDH, and INR and lower albumin, Hb, LDL cholesterol, total cholesterol and thrombocytes. SVS was absent in 27% of patients with active cancer (*n* = 28/89) and in 15% of those without active cancer (*n* = 176/1187) (*p* = 0.004). The absence of HVS did not differ between patients with active cancer (*n* = 30/77, 39%) and those without active cancer (*n* = 319/1004, 32%, *p* = 0.21). HVS was detected in 74% of patients with proximal occlusions (*n* = 680/913) and 31% of patients with distal occlusions (*n* = 51/167). In patients with cancer and distal occlusion only, 76% (*n* = 19/25) demonstrated present SVS and 10% (*n* = 1/10) present HVS. The Supplementary Table S2 summarized the characteristics differences between patients with and without SVS and patients with and without HVS, respectively. Besides the age at admission, time from last-known-well to admission, the proposition of ESUS, and levels of albumin and INR, there was no difference between SVS and HVS groups. Of patients who had both imaging modalities (native CT + MRI with SWI) available, and in whom IVT had not been administered in between (*n* = 77), 82% of those with no SVS (*n* = 14/17) also had no HVS. In contrast, only 47% of patients with no HVS (*n* = 14/30) also had no SVS (Supplementary Table S3).

### The absence of the susceptibility vessel sign and cancer-related stroke

**Figure 2:**
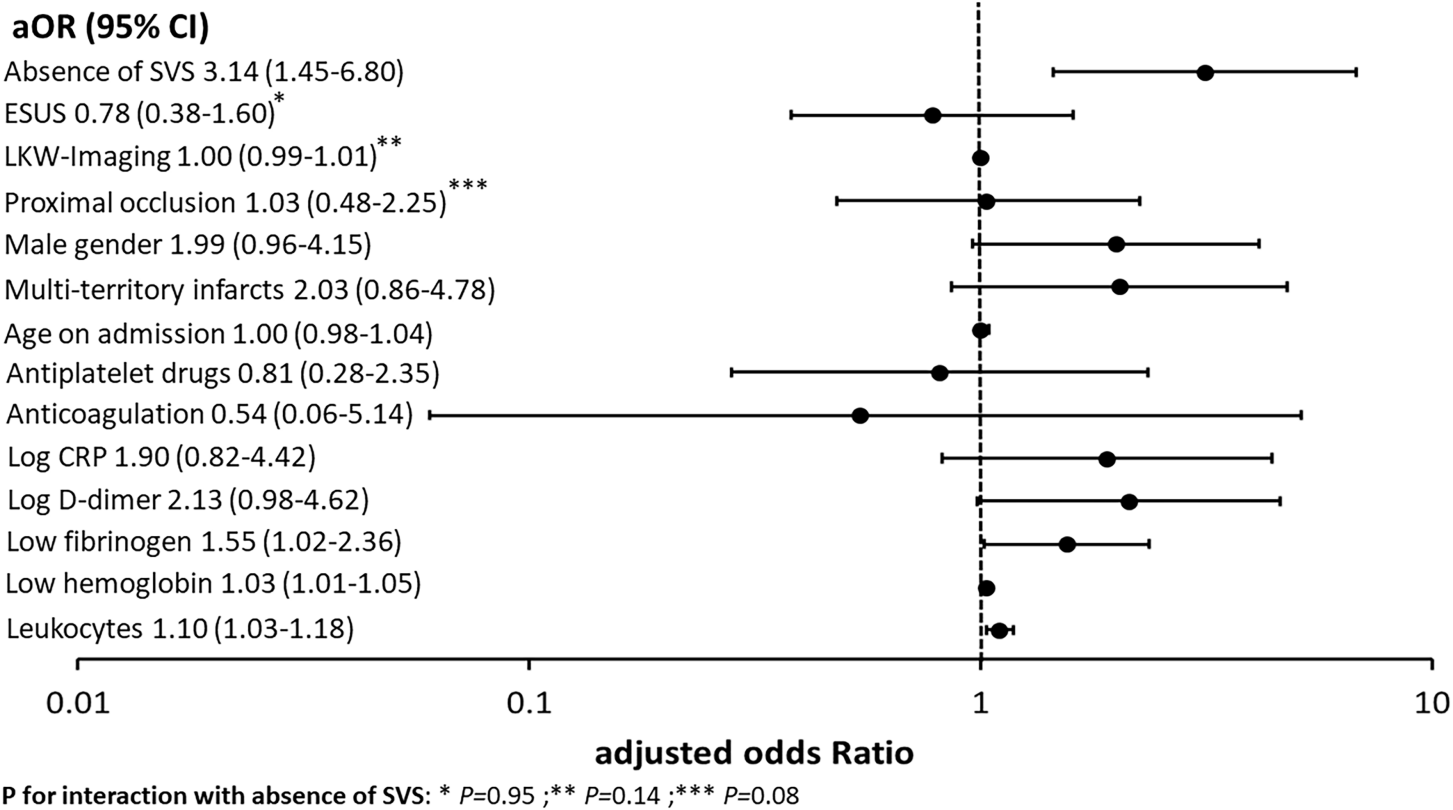
Association between active cancer, the absence of SVS, cancer-related imaging findings and blood biomarkers, and demographics in the multivariate logistic regression. This figure summarizes the association between active cancer, the absence of SVS and other preselected cancer-related biomarkers with corresponding adjusted odds ratios (aOR) and 95% confidence intervals (95% CI). The absence of SVS showed the strongest association with active cancer in this study cohort. CRP indicates C-reactive protein; ESUS, embolic stroke of undetermined source; LKW-imaging, time between last-known-well to imaging and SVS, susceptibility vessel sign.

In univariable analysis, the absence of SVS was associated with active cancer (OR 2.12, 95% CI 1.29–3.48). Figure 2 summarizes the results of the multivariable regression analyses. An association with active cancer was found for the absence of SVS (aOR 3.14, 95% CI 1.45–6.80), higher leukocyte counts in G/L (aOR 1.10, 95% CI 1.03–1.18), lower fibrinogen in g/L (aOR 1.55, 95% CI 1.02–2.36) and hemoglobin in g/L (aOR 1.03, 95% CI 1.01–1.05). The association between the absence of SVS and active cancer was not influenced by proximal occlusion (*p* for interaction = 0.08), the LKW-imaging time (*p* for interaction = 0.14) and ESUS (*p* for interaction = 0.95). Too few cases with active cancer were available to perform subgroup analyses of the different common stroke etiologies at discharge. Regarding the prediction of active cancer, the absence of SVS taken alone had a sensitivity of 27% (95% CI 18–37%), specificity of 85% (83–87%), PPV of 12% (8–17%), NPV of 94% (92–95%), LR+ of 1.82 (1.26–2.63) and LR− of 0.86 (0.75–0.98). The absence of SVS was not associated with occult cancer neither in univariable (OR 1.91, 95% CI 0.69–5.34) nor multivariable analysis (aOR 3.20, 95% CI 0.73–13.94). Subgroup analyses were not performed due to insufficient sample size.

### The absence of the hyperdense vessel sign and cancer-related stroke

In univariable and multivariable logistic regression analyses, no association between active cancer and absence of HVS was found (OR 1.37, 95% CI 0.85–2.21 and aOR 1.07, 95% CI 0.54–2.11, respectively). The results of the multivariable analyses are summarized in Supplementary Figure S4. No interaction was found with proximal occlusion (*p* = 0.99) or LKW-imaging time (*p* = 0.63). Despite positive interaction for ESUS (*p* = 0.04), the association between active cancer and the absence of HVS in the subgroup of patients with ESUS remained non-significant (aOR 2.03, 95% CI 0.40–10.17).

### Predictive value of SVS and HVS in the diagnosis of cancer-related stroke

The variables included in logistic regression analyses were reused to assess predictive models including or excluding SVS status and HVS status separately.

**Figure 3:**
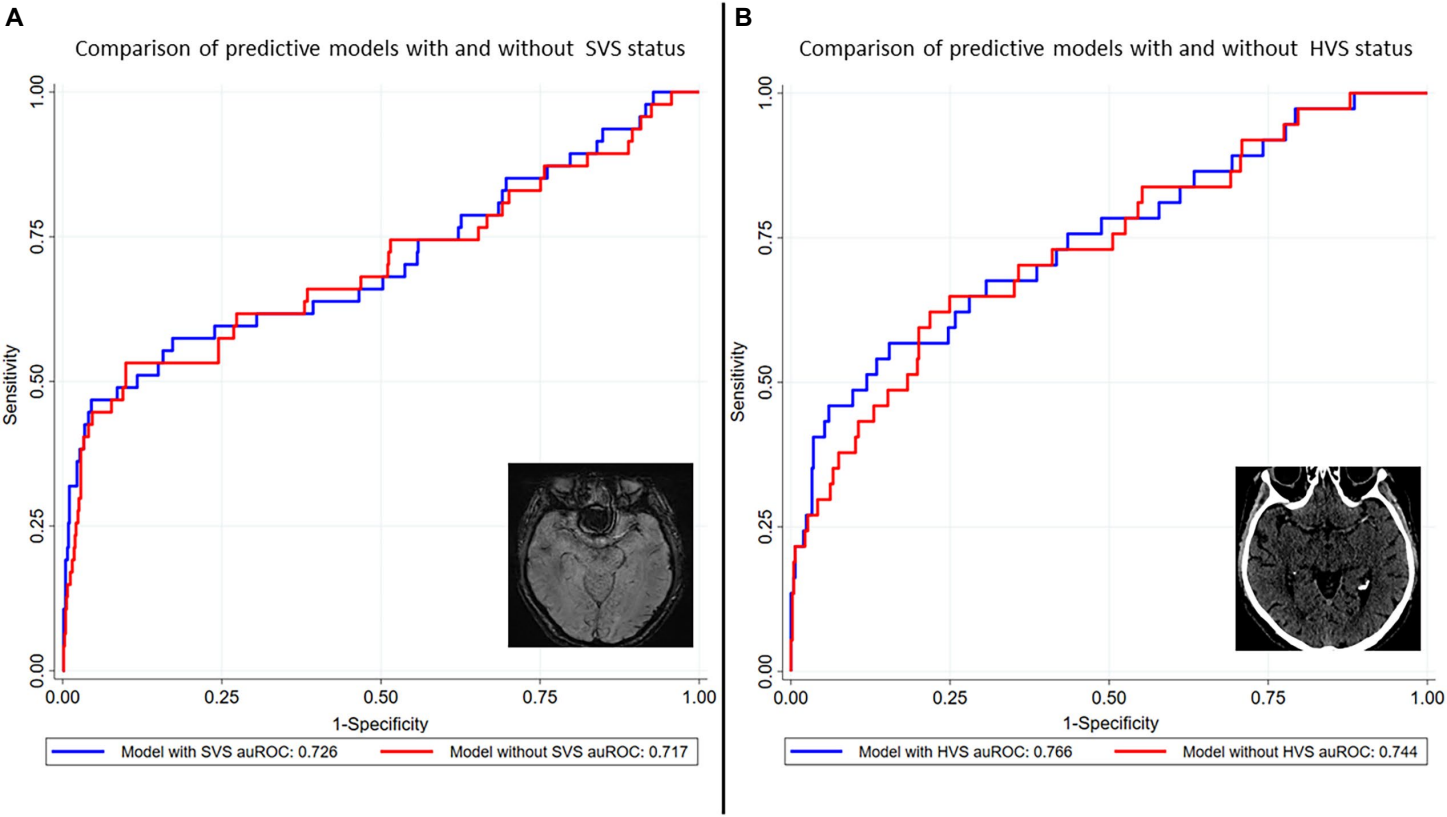
Predictive models for cancer-related stroke with and without SVS and HVS status. Models including and excluding SVS and HVS status, respectively, were developed using the covariates from logistic regression analyses (male gender, age at admission, prior treatment with anticoagulants and antiplatelet drugs, proximal occlusion, LKW-imaging, ESUS, multiterritory infarcts, CRP, D-dimer, fibrinogen, Hb and leukocytes). The auROCs of the models including and excluding SVS were 0.726 (95% CI 0.632–0.820) and 0.717 (95% CI 0.619–0.815), respectively **(A)**. According to the DeLong test, both models showed no significant difference (*p* = 0.73). The auROCs of the models including and excluding HVS were 0.766 (95% CI 0.672–0.860) and 0.744 (95% CI 0.650–0.838), respectively **(B)**. The DeLong test did not show a significant difference between both models (*p* = 0.41). auROC indicates area under the receiver operating characteristics curve; CRP, C-reactive protein; ESUS, embolic stroke of undetermined source; Hb, hemoglobin; HVS, hyperdense vessel sign; LKW-imaging, time between last-known-well to imaging; SVS, susceptibility vessel sign.

The auROCs of the models including and excluding SVS were 0.726 (95% CI 0.632–0.820) and 0.717 (95% CI 0.619–0.815), respectively (Figure 3A). According to the DeLong test, both models showed no significant difference (*p* = 0.73). The auROCs of the models including and excluding HVS were 0.766 (95% CI 0.672–0.860) and 0.744 (95% CI 0.650–0.838), respectively (Figure 3B). The DeLong test did not show a significant difference between both models (*p* = 0.41).

## Discussion

This study’s main findings are as follow: (1) The association between the absence of SVS at baseline and active cancer was confirmed in a large cohort of stroke patients, (2) absence of HVS showed no association with active or occult cancer, (3) the association between the absence of SVS and occult cancer did not reach statistical significance.

The confirmation of the association between the absence of SVS and active cancer in the overall stroke population will help to identify paraneoplastic hypercoagulability in the presence of underlying cancer. The optimal secondary prevention in cancer-related patients is still debated. Some studies support the use of anticoagulation over antiplatelet drugs (3). The TEACH Trial (pilot randomized trial) indicated the non-practicality of therapy with low molecular weight heparin in the long term (31). The use of oral anticoagulants seems more appropriate but major guidelines currently advocate for randomized trials (32, 33). According to previous evidence, the absence of SVS demonstrated the strongest association with active cancer in multivariable analysis (Figure 2) (14). Even if the auROC of the model with SVS tended to be greater, no statistical difference was found (Figure 3A). For this reason physicians must assess the absence of SVS in the clinical context by evaluating symptoms, other markers of cancer-related stroke and the presence of alternative causes of stroke before making a treatment decision (13). Like the presence of SVS, the presence of HVS is thought to indicate red blood cell-rich thrombi (16, 17, 34, 35). It is then commonly assumed that the absence of HVS corresponds to fibrin and platelet-rich thrombi (16). However, studies directly comparing HVS and SVS as surrogate marker for the microscopic composition of thrombi are scarce (16).

In our study, we could not demonstrate an association between the absence of HVS and active cancer. The predictive model including HVS performed slightly better than the model excluding HVS but was not statistically different (Figure 3B). One possible explanation for our results could be due to different sensitivities of SVS and HVS regarding the identification of intracranial occlusion. In line with the current evidence, SVS was more prevalent (84%) than HVS (68%) in the case of intracranial occlusion in our study (Supplementary Table S2) (36, 37). It is possible that HVS was absent because of lower sensitivity in intracranial occlusion detection rather than the presence of fibrin- and platelet-rich thrombus. In the literature, a positive HVS has been reported in 47% for proximal occlusions and 37% for distal occlusions (38). In our population, HVS was detected in 74% of patients with proximal occlusions (including basilar artery and vertebral artery) and in 31% of patients with distal occlusions. This discrepancy is most likely explained by the difference in slice thickness (1 mm in our population compared to a mean slice thickness of 1.65 to 4.5 mm), with the sensitivity of HVS increasing as the slice thickness decreases (38). There is no literature on the relevance of slice thickness of SWI regarding proximal or distal vessel occlusions. However, similar detection rates were observed in our population (73% in patients with active cancer and 85% in patients without active cancer) with SWI slice thickness of 1.6, 1.8 or 2.0 mm compared to studies with SWI slice thickness of 3 mm (79%) (39). The fact that the absence of SVS correlated more with the HVS status than the absence of HVS with the SVS status in the cases where intermodal comparison was available (*n* = 77) supports this hypothesis (Supplementary Table S2).

As HVS is considered time-dependent with loss of density over time and is more frequently seen in proximal occlusion, we performed a subgroup analysis (25, 26, 40). Nevertheless, no correlation of HVS-appearance with LKW-imaging time and proximal occlusion was found in our study cohort. The generalization of our previous findings in the context of occult cancer is inconclusive. It is estimated that occult cancer is present in 2–4% of the overall stroke population and this percentage can be as high as 10% in the subgroup of patients with undetermined stroke etiology (4–7, 41). The limited number of occult cancers in our study (1.6%), potentially due to the retrospective design, may be a possible explanation for our findings. Even if an interaction with ESUS was significant, the association between SVS and occult cancer remained statistically insignificant in the subgroup analysis. In patients with occult cancer, compared to patients with active known cancer, the impact of paraneoplastic hypercoagulability may be smaller as markers of coagulation (D-dimer) and inflammation (CRP and Hb) tend to be less altered at admission in the first group (20). This may influence the thrombus composition and consequently change the thrombus imaging characterization.

### Limitations

Firstly, our study was retrospective and included a largely homogeneous Swiss population, limiting the generalizability of the results and potentially underestimating the real incidence of occult cancer due to diagnoses made at other centers. Secondly, the local center followed an MRI-based acute stroke concept, which may have led to a selection bias regarding the association between the absence of HVS and active cancer. Further studies are needed, including patients with acute ischemic stroke principally diagnosed by CT and with available HVS status. Furthermore acute MRI is not routinely performed in all stroke centers. This could limit the generalization of our findings. Third, the lack of direct histological correlation to thrombus imaging characteristics limits the validation of the study hypothesis. Fourth, the variable use of 1.5 T and 3 T MRI could have influenced the determination of SVS status and, thus, the association between the absence of SVS and cancer (active or occult).

## Conclusion

This study confirmed the association between the absence of SVS and active cancer in all stroke patients. The LKW-imaging time, the site of occlusion and the absence of a common stroke etiology at discharge do not seem to play a role in this association. This study could not demonstrate an association between the absence of HVS and active cancer.

## Author’s note

MB reports research support from the “Kurt und Senta Hermann-Stiftung.” TM reports research support from the Bangerter Rhyner Foundation, Swiss National Foundation, and the Swiss Heart Foundation. HM reports personal consulting fees outside of this study from Servier, Bayer, Medtronic, Stryker and Cerenovus. EP reports grants from the Swiss National Science Foundation, unrelated to the submitted work. Mordasini reports receipt of research support from Siemens, Cerenovus, iSchmaview, Medtronic, Stryker, the Swiss Heart Foundation and the Swiss National Foundation, receipt of consultant fees payed to the institution from Medtronic, Cerenovus, Phenox and Microvention during the conduct of the study, unrelated to the submitted work. MA reports personal fees from Bayer, Bristol-Myers Squibb, Medtronic, Amgen, Daiichi Sankyo, Nestlé Health Sciences, Boehringer Ingelheim, and Covidien during the conduct of the study. UF reports grants during the conduct of the study from Medtronic, Stryker, and CSL Behring, unrelated to the submitted work. JK reports grants from the Swiss Academy of Medical Sciences/Bangerter Foundation, Swiss Stroke Society, and Clinical Trials Unit Bern during the conduct of the study. SJ reports grants from the Swiss National Science Foundation and the Swiss Heart Foundation.

## Data availability statement

The raw data supporting the conclusions of this article will be made available by the authors, without undue reservation.

## Ethics statement

The studies involving human participants were reviewed and approved by Kantonale Ethikkomission Bern. Written informed consent for participation was not required for this study in accordance with the national legislation and the institutional requirements.

## Author contributions

MB contributed to conception and design, data acquisition, analysis and interpretation of data, and writing of the publication. LG contributed to conception and design, data acquisition, critical revision of the manuscript for important intellectual content. JG, FS, LC and CK contributed to data acquisition and critical revision of the manuscript for important intellectual content. NB contributed to conception and design and critical revision of the manuscript for important intellectual content. SJ contributed to conception and design, critical revision of the publication for important intellectual content, and supervision. JK contributed to conception and design, analysis and interpretation of data, critical revision of the publication for important intellectual content, and supervision. All other authors contributed to interpretation of data and critical revision of the manuscript for important intellectual content.

## Funding

This work was supported by grants provided by the “Kurt und Senta Hermann-Stiftung” (grant number WNK-228), by the Swiss Academy of Medical Sciences (SAMS) within the framework of the Young Talents in Clinical Research Program (grant number YTCR 03/19) and grants provided by the Clinical Trials Unit Bern, University of Bern (grant number 84801869). Open access funding by the University Of Bern.

## Conflict of interest

The authors declare that the research was conducted in the absence of any commercial or financial relationships that could be construed as a potential conflict of interest.

## Publisher’s note

All claims expressed in this article are solely those of the authors and do not necessarily represent those of their affiliated organizations, or those of the publisher, the editors and the reviewers. Any product that may be evaluated in this article, or claim that may be made by its manufacturer, is not guaranteed or endorsed by the publisher.
