# Supplementary material for: Absence of susceptibility vessel sign and hyperdense vessel sign in patients with cancer-related stroke
Source: Front Neurol. 2023 Mar 20;14:1148152. doi: 10.3389/fneur.2023.1148152 (PMC10067593; doi:10.3389/fneur.2023.1148152)
Supplement: Supplementary file 1 [file Data_Sheet_1.docx]

**SUPPLEMENTAL MATERIALS**

**Absence of susceptibility vessel sign and hyperdense vessel sign in patients with cancer-related stroke.**

Morin Beyeler MD^1,2^*, Lorenz Grunder MD^3^*, Jayan Göcmen MS^1^, Fabienne Steinauer MS^1^, Nebiyat F. Belachew MD^4^, Moritz Kielkopf MD^1^, Leander Clénin MD^1^, Madeleine Mueller MD^1^, Norbert Silimon MD^1^, Christoph Kurmann MD^3^, Thomas Meinel MD^1^, Philipp Bücke MD^1^, David Seiffge MD^1^, Tomas Dobrocky MD^3^, Eike I Piechowiak MD^3^, Sara Pilgram-Pastor MD^3^, Heinrich P. Mattle MD^1^, Babak B. Navi MD, MS^5^, Marcel Arnold MD^1^, Urs Fischer MD^1,6^, Thomas Pabst MD^7^, Jan Gralla MD^3^, Martin D. Berger MD^7^, Simon Jung MD^1^* and Johannes Kaesmacher MD^3^*

* equal contribution

1) Department of Neurology, Inselspital, Bern University Hospital, and University of Bern, Switzerland

2) Graduate School for Health Sciences, University of Bern, Switzerland

3) Institute for Diagnostic and Interventional Neuroradiology, Inselspital, Bern University Hospital, and University of Bern, Switzerland

4) Department of Neuroradiology, University Hospital, Freiburg, Germany

5) Clinical and Translational Neuroscience Unit, Feil Family Brain and Mind Research Institute and Department of Neurology, Weill Cornell Medicine, New York, New York, USA

6) Neurology Department, University Hospital of Basel, University of Basel, Basel, Switzerland

7) Department of Medical Oncology, Inselspital, Bern University Hospital, and

University of Bern, Switzerland

**Supplementary Figures**


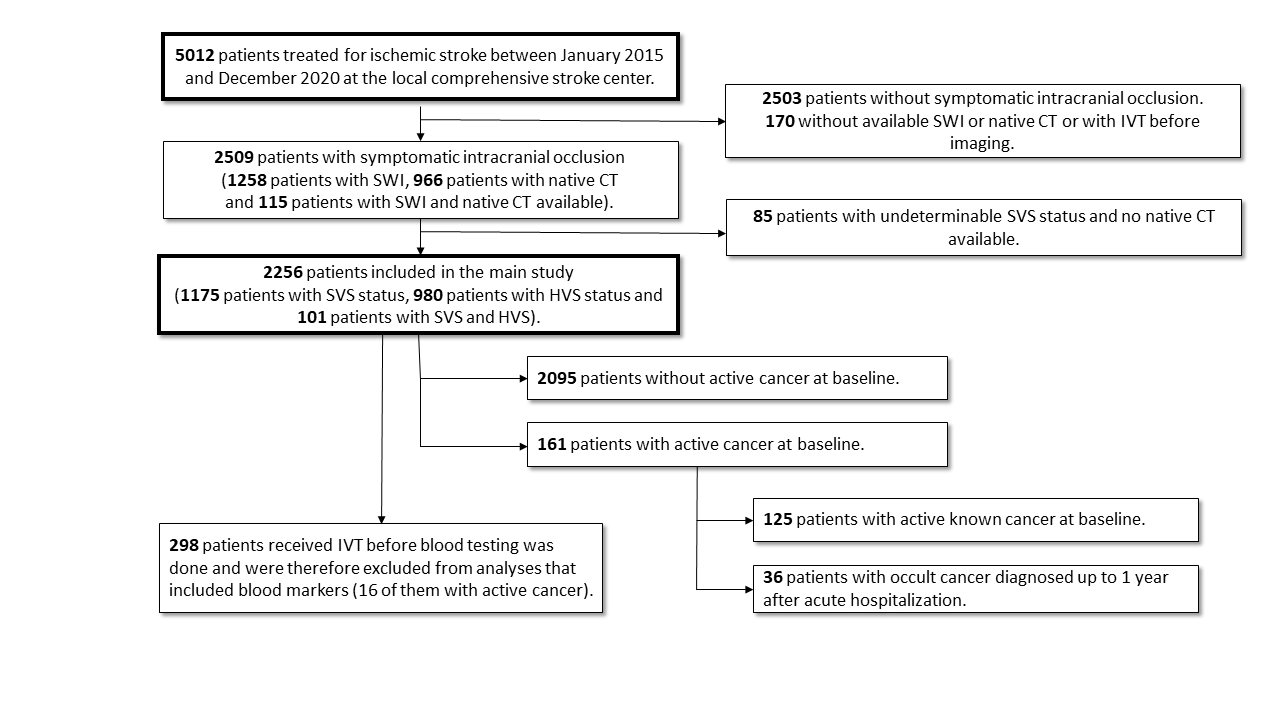


**eFigure I** – Study flowchart.
Inclusion and exclusion of study participants. HVS indicates hyperdense vessel sign; IVT, intravenous thrombolysis; native CT, native computed tomography; SVS, susceptibility vessel sign; and SWI susceptibility-weighted imaging.


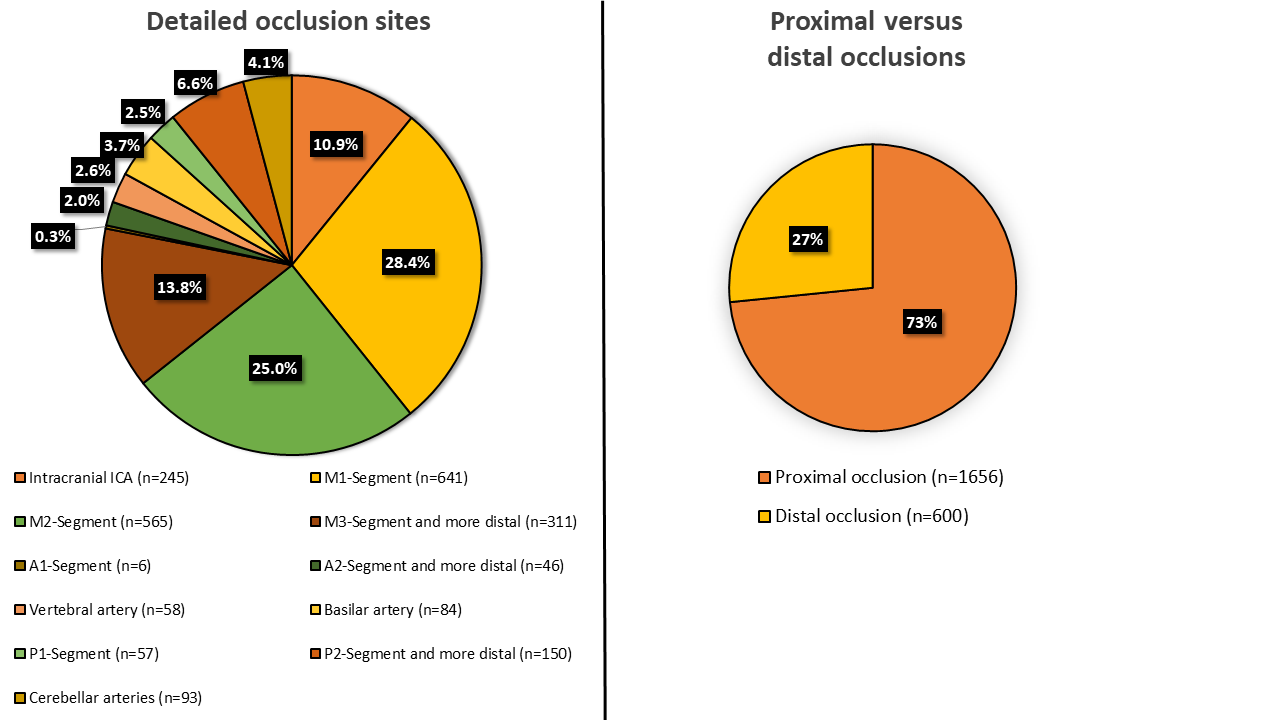


**eFigure II –** Distribution of occlusion sites in the study cohort.
The pie chart on the left shows details of the occlusion sites. The one on the right shows the proportions of proximal and distal strokes: Occlusions in ICA, M1, M2, A1, P1 and basilar artery were considered proximal occlusions.

A1-2-segments indicates the A-segments of the anterior cerebral artery; ICA, internal carotid artery; M1-2-3-segments, M-segments of the middle cerebral artery; and P1-2-segments, P-segments of the posterior cerebral artery.


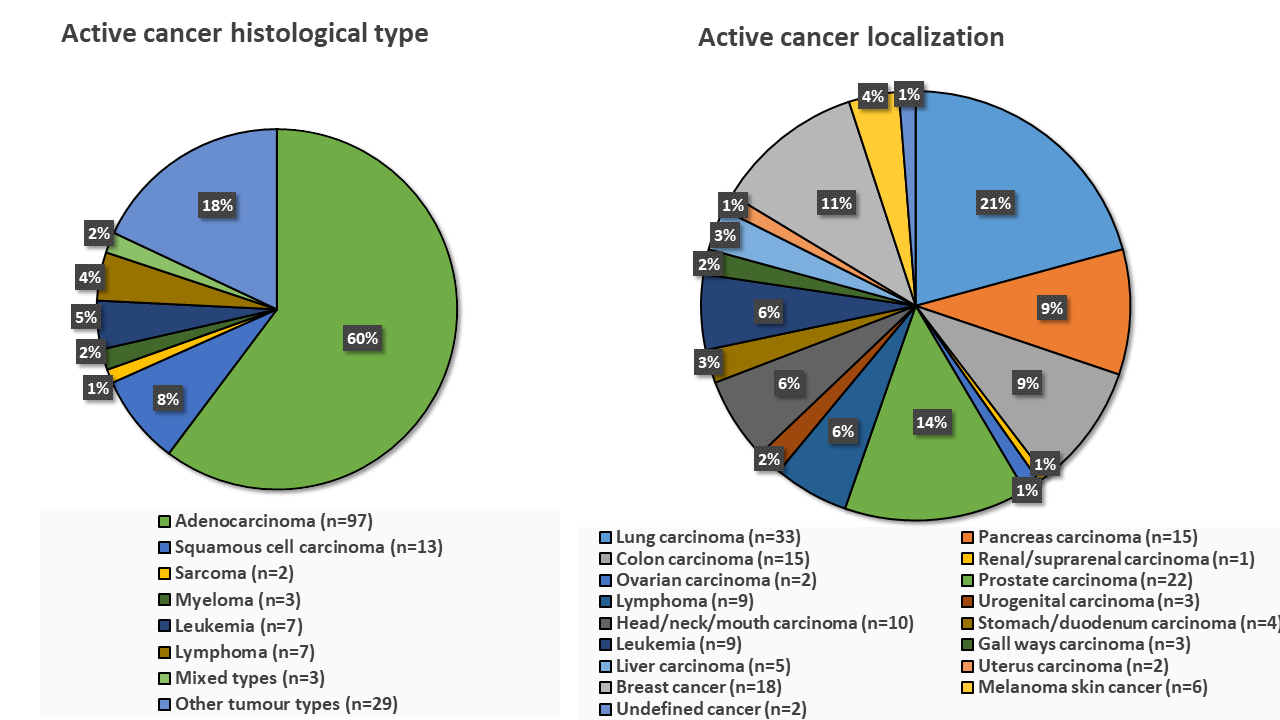

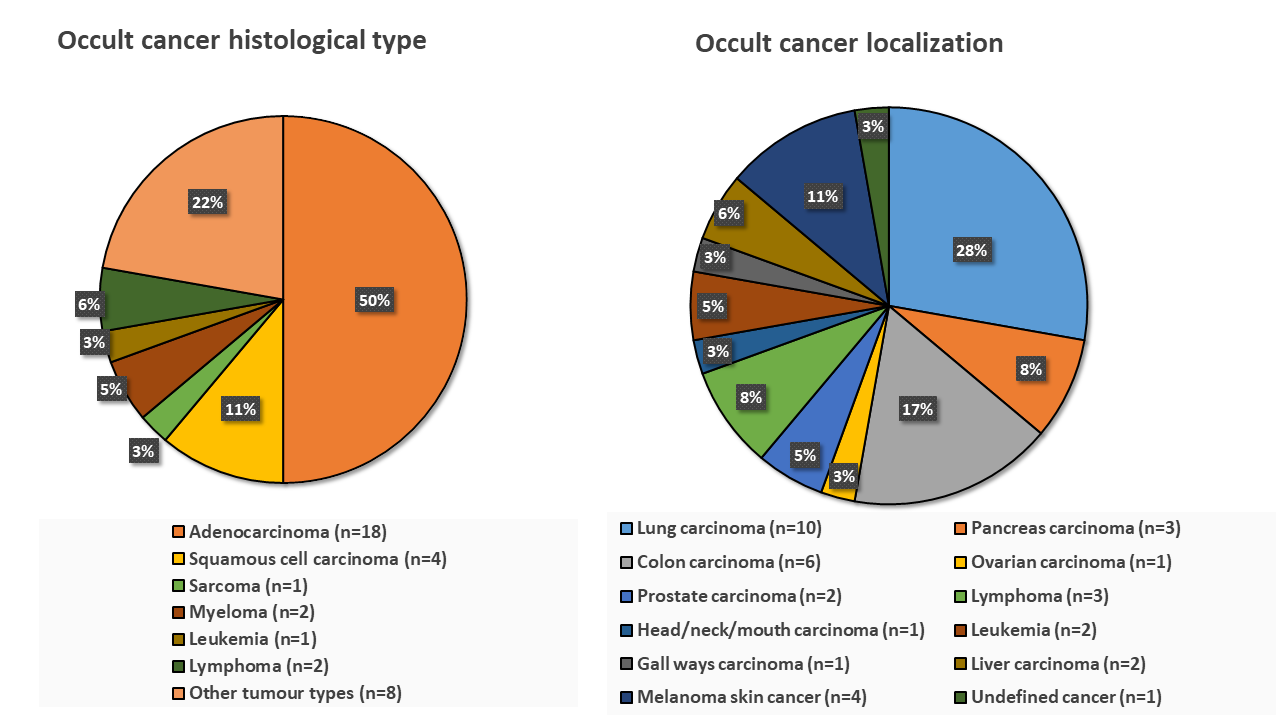


**eFigure III** – Histological characterizations and locations of active cancer and occult cancer.


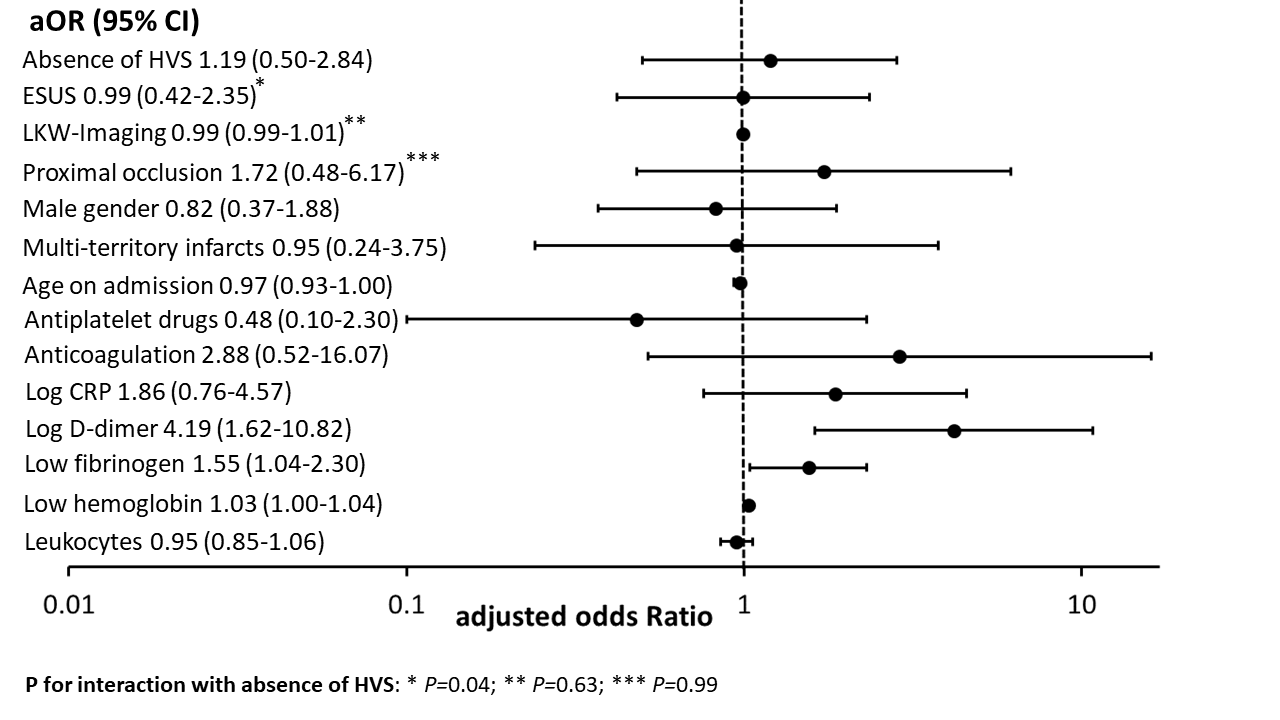


**eFigure IV –** Association between active cancer, absence of HVS, cancer-related imaging findings and blood biomarkers, and demographics in the multivariable logistic regression.

This figure summarizes the association between active cancer, the absence of HVS and other preselected cancer-related biomarkers with corresponding adjusted odds ratios (aOR) and 95% confidence intervals (95% CI). The absence of HVS was not associated with active cancer in our study cohort. The absence of HVs war associated with elevated D-dimer and low fibrinogen and hemoglobin. CRP indicates C-reactive protein; ESUS, embolic stroke of undetermined source; HVS, hyperdense vessel sign; and LKW–imaging, time from last-known-well to imaging.

|  | All patients  (N=2256) | No cancer  (N=2095) | Active cancer  (N=161) | p-value |
| --- | --- | --- | --- | --- |
| Baseline | | | | |
| Gender, male No. / total No. (%) | 1234/2256 (54.7) | 1147/2095 (54.7) | 87/161 (54.0) | 0.87 |
| Age at admission (median, IQR) | 73.7 (62.9–82) | 75.2 (63.7–83.7) | 74.2 (65.1–81.9) | 0.53 |
| Prestroke independence (mRS≤2), No. / total No. (%) | 1364/1552 (87.9) | 1284/1456 (88.2) | 80/96 (83.3) | 0.19 |
| Prior anticoagulation (vitamin K-antagonist and NOAC), No. / total No. (%) | 400/2024 (19.8) | 336/1880 (17.9) | 64/144 (44.4) | <0.001 |
| Prior antiplatelet drugs, No. / total No. (%) | 655/2022 (32.4) | 621/1878 (33.1) | 34/144 (23.6) | 0.021 |
| Lipid Lowering drugs pre-stroke, No. / total No. (%) | 584/2017 (29.9) | 539/1873 (28.8) | 45/144 (31.3) | 0.57 |
| Risk factors | | | | |
| Previous stroke, No. / total No. (%) | 302/2033 (14.9) | 284/1888 (15) | 18/145 (12.4) | 0.47 |
| Hypertension, No. / total No. (%) | 1490/2031 (73.4) | 1380/1886 (73.2) | 110/142 (75.9) | 0.56 |
| Diabetes, No. / total No. (%) | 375/2025 (18.5) | 350/1882 (18.6) | 25/143 (17.5) | 0.82 |
| Hyperlipidemia, No. / total No. (%) | 1308/2016 (64.9) | 1225/1873 (65.4) | 83/143 (58.0) | 0.084 |
| Smoking (history or current), No. / total No. (%) | 386/1892 (20.4) | 351/1758 (20) | 35/99 (26.1) | 0.095 |
| Coronary artery disease, No. / total No. (%) | 367/2023 (18.1) | 347/1879 (18.5) | 20/144 (13.9) | 0.18 |
| Stroke characteristics | | | | |
| NIHSS on admission, (median, IQR) | 9 (3–17) | 9 (3–17) | 9 (4–17) | 0.49 |
| Time from last-known-well to admission in min. (median, IQR) | 185 (93–486) | 185 (93–483) | 185 (95–524) | 0.75 |
| Time from last-known-well to imaging in min. (median, IQR) | 178 (103–435) | 180 (103–433) | 156 (93–538) | 0.53 |
| IVT, No. / total No. (%): | 809/2034 (39.8) | 773/1888 (40.9) | 36/146 (24.7) | <0.001 |
| MRI on admission, No. / total No. (%): | 1015/1879 (54) | 951/1750 (54.3) | 64/129 (49.6) | 0.31 |
| **Site of occlusion,** No. / total No. (%): | | | | |
| Intracranial carotid artery | 245/2255 (10.9) | 233/2094 (11.1) | 12/161 (7.5) | 0.007 |
| M1 Segment of MCA | 641/2255 (28.4) | 588/2094 (28.1) | 53/161 (32.9) |  |
| M2 Segment of MCA | 565/2255 (25) | 512/2094 (24.4) | 53/161 (32.9) |  |
| Other occlusion in the anterior circulation | 363/2256 (16.1) | 338/2094 (16.1) | 25/161 (15.5) |  |
| Occlusion in the posterior circulation | 442/2256 (19.6) | 424/2094 (20.2) | 18/161 (11.2) |  |
| Proximal occlusion | 1655/2255 (73.4) | 1529/2094 (73.0) | 126/161 (78.3) | 0.17 |
| **Stroke etiology (TOAST)**  No. / total No. (%): | | | | |
| Cardioembolic | 880/2256 (39) | 823/2095 (39.3) | 57/161 (27.5) | 0.16 |
| Small-vessel occlusion | 0/2256 (0) | 0/2095 (0) | 0/161 (0) |  |
| Large-artery atherosclerosis | 243/2256 (10.8) | 232/2095 (11.1) | 11/161 (6.8) |  |
| Stroke of other determined etiology | 93/2256 (4.1) | 86/2095 (4.1) | 7/161 (4.3) |  |
| Stroke of undetermined etiology | 1040/2256 (46.1) | 954/2095 (45.5) | 86/161 (53.4) |  |
| ESUS, No. / total No. (%) | 1011/2255 (44.8) | 929/2094 (44.4) | 82/161 (50.9) | 0.12 |
| Multi-territory infarcts, No. / total No. (%) | 214/2255 (9.5) | 185/2094 (8.8) | 29/161 (18.0) | <0.001 |
| Absence of SVS, No. / total No. (%) | 200/1276 (15.7) | 176/1187 (14.8) | 24/89 (27) | 0.004 |
| Absence of HVS, No. / total No. (%) | 349/1081 (32.3) | 319/1004 (31.8) | 30/77 (39) | 0.21 |
| Baseline laboratory findings | | | | |
| Glucose in mmol/L (median, IQR) | 6.5 (5.8–7.8) | 6.5 (5.8–7.8) | 6.45 (5.6–7.6) | 0.60 |
| LDL cholesterol in mmol/L  (median, IQR) | 2.52 (1.88-3.26) | 2.55 (1.89-3.29) | 2.15 (1.74-2.71) | <0.001 |
| Total cholesterol in mmol/L (median, IQR) | 4.57 (3.78-5.37) | 4.6 (3.8-5.4) | 4.17 (3.46-4.95) | <0.001 |
| Albumin in g/L (median, IQR) | 31.8 (26-35) | 32 (27-36) | 27 (22-34) | 0.003 |
| LDH in U/L (median, IQR) | 412.5 (350.5-508) | 407 (348-498) | 500 (399-695) | <0.001 |
| D-dimer in µg/L (median, IQR) | 993 (500–2204) | 965.5 (482–2011.5) | 2226 (962–8009) | <0.001 |
| Hemoglobin in g/L (median, IQR) | 136 (122–147) | 137 (123–147) | 122 (103-136) | <0.001 |
| C-reactive protein in mg/L (median, IQR) | 4 (2–11) | 3 (3–10) | 12 (4–42) | <0.001 |
| Leukocytes in G/L (median, IQR) | 8.4 (6.77–10.6) | 8.39 (6.8–10.5) | 8.98 (6.9–12) | 0.081 |
| Thrombocytes in G/L (median, IQR) | 222 (180–268) | 223 (183-269) | 200 (161–262) | 0.004 |
| Fibrinogen in g/L (median, IQR) | 3.12 (2.62–3.79) | 3.12 (2.63–3.8) | 3.04 (2.42–3.88) | 0.29 |
| INR (median, IQR) | 1.02 (0.98–1.1) | 1.02 (0.98–1.1) | 1.7 (1–1.21) | <0.001 |
| ESUS indicates embolic stroke of undetermined source; HVS, hyperdense vessel sign; INR, International Normalized Ratio; IQR, interquartile range; IVT, intravenous thrombolysis; LDH, lactate dehydrogenase; LDL, low-density lipoprotein; MRI, magnetic resonance imaging; MCA, middle cerebral artery; NIHSS, National Institutes of Health Stroke Scale; NOAC, non-vitamin K antagonist oral anticoagulant; SVS, susceptibility vessel sign; TOAST, Trial of ORG 10172 in Acute Stroke Treatment. | | | | |

**Supplementary table I** – Comparison of baseline characteristics between patients with active cancer versus no cancer.

|  | SVS **⊕**  (N=1076) | SVS **⊖**  (N=200) | p-value | HVS **⊕**  (N=732) | HVS **⊖**  (N=349) | p-value |
| --- | --- | --- | --- | --- | --- | --- |
| Baseline | | | | | | |
| Gender, male No. / total No. (%) | 599/1076 (54.7) | 112/200 (56.0) | 0.94 | 386/732 (52.7) | 190/349 (54.4) | 0.6 |
| Age at admission (median, IQR) | 73.1 (62.4-82) | 74.2 (62-3–81.5) | 1.0 | 78.4 (67.55-85.05) | 75.8 (64.9-83.8) | 0.019 |
| Active malignancy No. / total No. (%) | 65/1076 (6) | 24/200 (12) | 0.004 | 47/732 (6.4) | 30/349 (8.6) | 0.21 |
| Prior anticoagulation (vitamin K-antagonist and NOAC), No. / total No. (%) | 128/954 (13.4) | 45/162 (27.8) | <0.001 | 152/682 (22.3) | 90/320 (28.1) | 0.048 |
| Prior antiplatelet drugs, No. / total No. (%) | 285/952 (29.9) | 56/162 (34.6) | 0.27 | 239/682 (35) | 111/320 (34.7) | 0.94 |
| Lipid Lowering drugs pre-stroke, No. / total No. (%) | 237/950 (24.9) | 63/161 (39.1) | <0.001 | 200/680 (29.4) | 205/115 (35.9) | 0.041 |
| Stroke characteristics | | | | | | |
| NIHSS on admission, (median, IQR) | 6 (2-12) | 4 (1-9) | <0.001 | 16 (9-21) | 10 (4-18) | <0.001 |
| Time from last-known-well to admission in min. (median, IQR) | 191 (89–549) | 280 (97–746) | 0.014 | 178 (108-332) | 187 (101-409) | 0.54 |
| Time from last-known-well to imaging in min. (median, IQR) | 197 (108–510) | 206 (106–552) | 0.61 | 159 (100-336) | 159 (106-376) | 0.57 |
| Proximal occlusion | 731/1076 (67.9) | 99/200 (49.5) | <0.001 | 680/731 (93) | 233/349 (66.8) | <0.001 |
| Stroke etiology (TOAST) No. / total No. (%): | | | | | | |
| Cardioembolic | 385/1076 (35.8) | 53/200 (27.5) | 0.041 | 318/732 (43.4) | 158/349 (45.3) | 0.33 |
| Large-artery atherosclerosis | 110/1076 (10.2) | 21/200 (10.5) |  | 87/732 (11.9) | 35/349 (10) |  |
| Stroke of other determined etiology | 56/1076 (5.2) | 8/200 (4) |  | 28/732 (3.8) | 7/349 (73.0) |  |
| Stroke of undetermined etiology | 525/1076 (48.8) | 118/200 (59) |  | 299/732 (40.8) | 149/349 (42.7) |  |
| ESUS, No. / total No. (%) | 508/1075 (47.3) | 114/20 (50.9) | 0.014 | 296/732 (40.4) | 143/349 (41) | 0.89 |
| Baseline laboratory findings | | | | | | |
| Albumin in g/L (median, IQR) | 32 (27-37) | 34 (28-37) | 0.44 | 32 (26-35) | 28 (22-35) | 0.032 |
| LDH in U/L (median, IQR) | 399 (347-480-5) | 432.5 (363-547) | 0.002 | 423 (359-509) | 452.5 (360-576) | 0.018 |
| INR (median, IQR) | 1.01 (0.97–1.07) | 1.03 (0.98–1.1) | 0.008 | 1.05 (1-1.12) | 1.03 (1-1.19) | 0.52 |
| ESUS indicates embolic stroke of undetermined source; HVS, hyperdense vessel sign; INR, International Normalized Ratio; IQR, interquartile range; LDH, lactate dehydrogenase; NIHSS, National Institutes of Health Stroke Scale; NOAC, non-vitamin K antagonist oral anticoagulant; SVS, susceptibility vessel sign; TOAST, Trial of ORG 10172 in Acute Stroke Treatment. | | | | | | |

**Supplementary table II** – Comparison of baseline characteristics between patients with and without susceptibility vessel sign (left part) and patients with and without hyperdense vessel sign (right part).


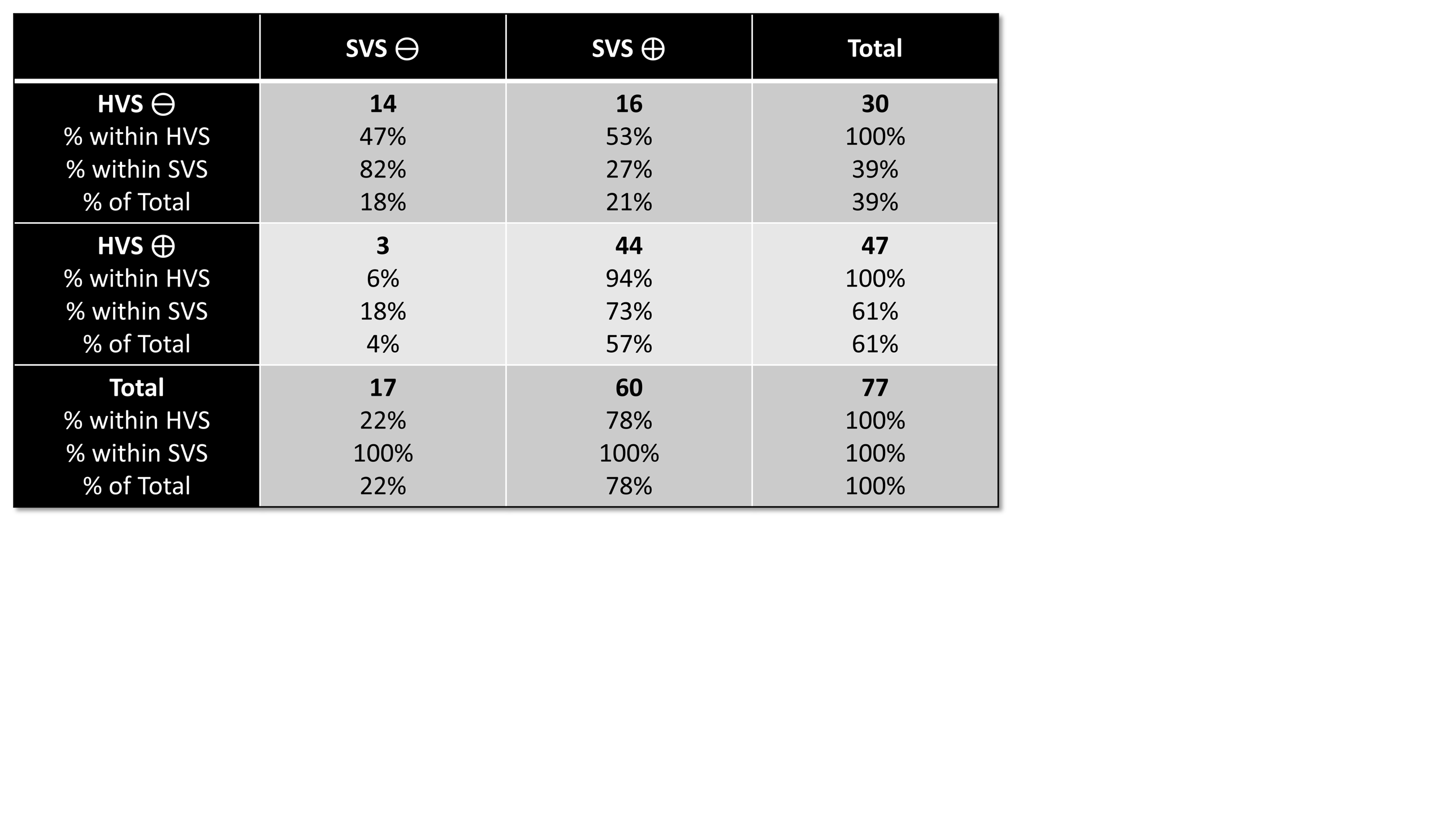


**Supplementary table III** – Cross tabulation with absolute numbers and percentages of patients with intracranial occlusion diagnosed on native CT + MRI with SWI at baseline and assessable SVS + HVS status. Patients treated with IVT before the assessed imaging were excluded.
HVS indicates hyperdense vessel sign; native CT, native computed tomography; SVS, susceptibility vessel sign; and SWI susceptibility-weighted imaging.
